# Specific recommendations (or lack thereof) for older patients with cardiovascular disease in the current European Society of Cardiology guidelines: From the Dutch Working Group of Geriatric Cardiology of the Netherlands Society of Cardiology (NVVC) and Special Interest Group Geriatric Cardiology of the Netherlands Society for Clinical Geriatrics (NVKG)

**DOI:** 10.1007/s12471-022-01674-y

**Published:** 2022-03-31

**Authors:** Kirsten. Boerlage-van Dijk, Charlotte E. P. Siegers, Noëmie T. A. E. Wouters, Miriam C. Faes, Robert A. M. Verbunt, J. Hans Geertman, Mieke van den Heuvel, Chajja T. U. van de Meerendonk, Su-San Liem, Jose P. Henriques, Jan Paul Ottervanger

**Affiliations:** 1 Department of Cardiology, OLVG, Amsterdam, The Netherlands; 2 Department of Cardiology, Amphia, Breda, The Netherlands; 3 Department of Geriatric Medicine, Amphia, Breda, The Netherlands; 4 Department of Cardiology, Maxima Medical Centre, Eindhoven, The Netherlands; 5 Department of Cardiology, Isala klinieken, Zwolle, The Netherlands; 6 Department of Cardiology, Medisch Spectrum Twente, Enschede, The Netherlands; 7 Department of Cardiology, Maasstad Hospital, Rotterdam, The Netherlands; 8 Department of Cardiology, Amstelland Hospital, Amstelveen, The Netherlands; 9 Department of Cardiology, Amsterdam University Medical Centre, Amsterdam, The Netherlands

**Keywords:** Older patients, ESC guidelines, Cardiovascular disease, Shared decision making, Palliative care, Advanced care planning

## Abstract

Due to population ageing, the number of older and frail patients with cardiovascular disease is increasing. In the current guidelines of the European Society of Cardiology specific recommendations for this older population are missing or scarce, probably due to limited evidence concerning diagnosis and treatment of cardiovascular disease in older patients. Moreover, recommendations on shared decision making, palliative care and advanced care planning are also essential in these guidelines. In this article we evaluate the current European of Society of Cardiology guidelines (2013–2020) to determine whether specific recommendations for older patients have been included.

## Introduction

In the next decades, progressive growth in the number of older persons (≥65 years) is expected. According to data from the United Nations, one in four persons in Europe and Northern America is expected to be ≥65 years old by 2050 [1]. As a result of population ageing, the number of older and frail persons with cardiovascular disease (CVD) is increasing. When treating CVD in older patients, physicians will face dilemmas due to comorbidities, polypharmacy, cognitive impairment and limited life expectancy. Scientific data on CVD in older persons with comorbidities are relatively scarce. Results from a younger trial population cannot be extrapolated to older patients. Hence, we are missing specific recommendations for older and frail CVD patients, where there is a growing need for evidence-based practice in this growing population to address the risk/benefit decisions we face in our daily practice. Although the terms frail and frailty are widely used in scientific studies there is no international consensus on definition or screening instrument [2]. Because the elderly are a very heterogeneous population reaching any such consensus can be quite difficult. Moreover, there is a variety of established screening instruments based on either functional status (Clinical Frailty Scale) or phenotype (Fried Frailty Phenotype Criteria) [3, 4]. Furthermore, other screening instruments such as PRISMA‑7 (brief auto-questionnaire) maybe useful [5]. However, none of these scales are mentioned in the guidelines.

The older patient is increasingly addressed in recent international guidelines and additional efforts are made by dedicated working groups. The European Society of Cardiology (ESC) has recently launched the ‘Task Force on Geriatric Cardiology’. Furthermore, specific guidelines and recommendations for geriatric cardiology have been published in Brazil and Spain recently [6, 7]. In addition, recommendations on shared decision making (SDM), palliative care and advanced care planning are essential. Older patients with multiple chronic conditions might have other goals and expectations of care and treatment than younger patients. Therefore, SDM is recommended for the integration of preferences and values of patients. All ESC guidelines briefly mention the individual responsibility of health professionals to make a final decision concerning the individual patient by facilitating consultation with the patient and caregiver in the ‘preamble’. Also, more and more guidelines contain additional recommendations about SDM in specific subchapters. To guarantee the best possible quality of life in end stages of cardiovascular disease, knowledge about palliative care is needed. Implementation of advance care planning is necessary to determine the overall goal of medical care and the interventions that should and should not be provided in the final stages of life.

In this article, we evaluate the current ESC guidelines (2013–2020) to determine whether they include specific recommendations for older patients.

## Recommendations for the older population in the ESC guidelines

Due to the limited length and readability of this manuscript we focussed on the most commonly used guidelines in clinical practice. We do provide a table (Tab. 1) that displays all the current ESC guidelines except “Management of CVD during pregnancy (2018)” [8]. This table provides information on whether the guidelines contain specific recommendations for older CVD patients. In the more recent (<5 years) guidelines older patients gained notably more attention compared with older guidelines.

**Table 1:** ESC guidelines and specific recommendations for older patients

Guidelines	Publication year	Dedicated (sub)chapter	Definition of older patient	Age specified	Gaps in evidence as identified by the guideline
Cardiac pacing/CRT	2013	No	No		No
Hypertrophic cardiomyopathy	2014	No	No		No
Non-cardiac surgery	2014	No	No		No
Aortic disease	2014	No	No		No
Infective endocarditis	2015	No	No		No
Pericardial disease	2015	Yes^a^	No		No
Pulmonary hypertension	2015	No	No		No
Ventricular arrythmia/SCD prevention	2015	Yes	No		No
Acute and chronic heart failure	2016	Yes	No		Yes
CVD prevention	2016	Yes	Yes	>65 yr	Yes
Valvular heart disease	2017	Yes	No		No
ST-segment elevation	2017	Yes	No		No
Peripheral arterial disease	2017	No	No		No
Arterial hypertension	2018	Yes	Yes	>65 yr	Yes
Myocardial revascularisation	2018	No	No		No
Syncope	2018	Yes	Yes	>60 yr	No
Acute pulmonary embolism	2019	No	No		No
Supraventricular tachycardia	2019	No	No		No
Dyslipidaemias	2019	Yes	Yes	>65 yr	Yes
Diabetes, pre-diabetes and CVD	2019	No	Yes	>65 yr	Yes
Chronic coronary syndromes	2019	Yes	Yes	>75 yr	No
ACS without persistent ST-segment elevation	2020	Yes	No		Yes
Atrial fibrillation	2020	Yes	No		No
Sports cardiology & exercise	2020	Yes	Yes	>65 yr	No
Adult congenital heart disease	2020	No	No		No^b^

Gaps in evidence that are specified in the referring guideline

*ACS* acute coronary syndromes, *CRT* cardiac resynchronisation therapy, *CVD* cardiovascular disease, *ESC* European Society of Cardiology, *SCD* sudden cardiac death

^a^Subchapter with limited recommendations, lack of evidence is highlighted

^b^Authors refer to the American Heart Association’s scientific statement: Congenital heart disease in the older adult: Circulation 2015

## Atrial fibrillation

The ESC atrial fibrillation (AF) guideline is a recent guideline with specific recommendations for older patients [9]. It addresses the evidence for the use of oral anticoagulation (OAC) and non-vitamin K antagonist oral anticoagulant (NOAC) in this population. Observational studies report a protective role of OAC in the prevention of cognitive impairment in AF patients, with ongoing RCTs on this subject. The guideline also reviews the insufficient evidence for rate versus rhythm control in older patients. Systematic ECG screening should be considered to detect AF in individuals ≥75 years or in those at high risk of stroke. Opportunistic screening of AF in older persons is briefly mentioned and seems cost-effective. Recommendations on patient involvement and shared decision making are provided.

## Arterial hypertension

The guideline on arterial hypertension provides a full chapter dedicated to hypertension in older patients (≥65 years) [10]. It mentions the evidence for antihypertensive treatment in older (≥65 years) and very old patients (≥80 years), with significant reduction of cardiovascular morbidity and all-cause mortality. Furthermore, the difficulty of treating hypertension in older patients is addressed. Recommendations for initiating antihypertensive treatment in older and very old patients and target blood pressures are provided. The guideline suggests close monitoring of antihypertensive therapy and its side effects for this specific population. There is a lack of evidence for treatment of frail, functionally dependent older patients and those with orthostatic hypotension (the definition of frail or frailty is not provided). A subchapter mentions the evidence for treatment of grade 1 hypertension in older patients, although this was studied in a relatively fit, independent older population. In the section ‘Gaps in the evidence and need for further studies’ it states that more data on the benefits of blood pressure treatment in very old patients and the impact of frailty are required.

## Pacing and cardiac resynchronisation therapy

The ESC guideline on cardiac pacing and cardiac resynchronisation therapy dates from 2013 [11]. Although pacemaker implantation occurs more frequently in the older population [12], specific recommendations for this population are lacking.

## Chronic coronary syndrome/acute coronary syndrome

The guideline on chronic coronary syndromes (CCS) provides a subchapter on older patients [13]. It discusses the high incidence and prevalence of coronary artery disease in older persons with the often atypical presentation and difficulties of treatment in this population. A table with recommendations for older patients with CCS is provided. The aspect of shared decision making has been identified as key in deciding between revascularisation and conservative treatment.

The recent non-ST-elevation acute coronary syndrome guideline recommends using the same diagnostic and interventional strategies for older patients as are being used for younger patients [14]. In frail elderly, a careful risk assessment should guide the decision for interventional versus conservative treatment. Furthermore, the use of antithrombotic agents and secondary prevention should be adapted to renal function and specific contraindications.

The guideline for ST-elevation myocardial infarction states that there is no upper limit of age for performing primary percutaneous coronary intervention [15].

## Heart failure

The heart failure (HF) guideline addresses the difficulty of diagnosing HF, specifically HF with preserved ejection fraction, in older patients with comorbidities [16]. Concerning HF medication, digitalis should be used with caution in older patients, especially in case of renal disease. Co-existing problems in the HF population are reviewed. Because frailty has implications for HF management, the guideline provides screening instruments for frailty in HF. Specific recommendations for elderly are provided, such as monitoring frailty, identifying reversible causes of deterioration in frailty score, and performing a medication review. Essential components of palliative care service are provided, including advance care planning. Furthermore, the guideline lists key topics on shared decision making, patient education and self-care.

In 2019, an ESC position paper on frailty in patients with HF was published [2]. The main goals were identifying a consensus definition of frailty, highlighting the importance of assessment of frailty and exploring the main domains of the Heart Failure Association Frailty Score (HFA frailty score). This score is specifically designed and validated to identify frailty in the HF population. The authors state that a routine assessment for frailty in HF patients should be used.

An additional ESC position paper on palliative care in HF was published in 2020 [17]. Five main recommendations and goals were provided regarding in whom, by whom and how palliative care should be delivered.

## Valvular heart disease

The guideline for valvular heart disease provides a subchapter about considerations in older patients [18]. Assessment of frailty is a key component as frailty is associated with increased morbidity and mortality after surgery and transcatheter aortic valve replacement (TAVR). Euro SCORE II is primarily used for risk stratification. The decision for surgical aortic valve replacement (SAVR) versus TAVR in patients with symptomatic aortic stenosis is evaluated with specific considerations for the Heart Team. In older patients with symptomatic mitral stenosis and high surgical risk, percutaneous mitral commissurotomy is advised, even if only in palliative setting. In this guideline, shared decision making or patient involvement are only mentioned briefly.

## Diabetes mellitus and dyslipidaemia

The recent diabetes guideline provides a short paragraph on less strict Hb1AC goals in older patients (≥65 year) with certain characteristics [19]. Recommendations on SDM are mentioned. The dyslipidaemia guideline contains a chapter dedicated to older patients [20]. Recommendations about primary prevention and adverse effects and interaction of statins are presented. Furthermore, the risk of statin-induced myopathy is increased in the older persons.

## Discussion and conclusion

The ESC guidelines in general provide limited recommendations for older patients, which is probably attributable to the limited evidence concerning diagnosis and treatment of CVD in older patients. However, older patients are increasingly addressed in more recently published guidelines. If recommendations are available often a definition of older patients is lacking. When mentioned, the definition is usually ≥65 years. Patients ≥65 years are currently represented in clinical trials, but beyond the age of 75 years data are often lacking. We think that it would be useful to provide specific recommendations for patients ≥65 years and ≥75 years in the guidelines. Due to the heterogeneity of the older population it might be wise to provide recommendations for specific subgroups of older patients, such as those with and without significant comorbidity or frailty, instead of recommendations for older persons in general. These recommendations should also include the impact of shared decision making, palliative care and advance care planning.

We do believe there is increasing awareness of older patients in clinical trials, conferences and daily practice. Hopefully, this will result in additional recommendations or, eventually, a guideline specifically designed for the older patient with CVD.

## References

[1] United Nations World Population Ageing 2019. https://www.un.org/en/development/desa/population/publications/pdf/ageing/WorldPopulationAgeing2019-Reportpdf. Accessed 23 Jan 2021.

[2] Vitale C, Jankowska E, Hill L (2019). Heart Failure Association/European Society of Cardiology position paper on frailty in patients with heart failure. Eur J Heart Fail.

[3] Fried LP, Tangen CM, Walston J (2001). Frailty in older adults: evidence for a phenotype. J Gerontol A Biol Sci Med Sci.

[4] Rockwood K, Andrew M, Mitnitski A (2007). A comparison of two approaches to measuring frailty in elderly people. J Gerontol A Biol Sci Med Sci.

[5] Raiche M, Hebert R, Dubois MF (2008). PRISMA-7: a case-finding tool to identify older adults with moderate to severe disabilities. Arch Gerontol Geriatr.

[6] Diez-Villanueva P, Ariza-Sole A, Vidan MT (2019). Recommendations of the geriatric cardiology section of the Spanish Society of Cardiology for the assessment of frailty in elderly patients with heart disease. Rev Esp Cardiol.

[7] Feitosa-Filho GS, Peixoto JM, Pinheiro JES (2019). Updated geriatric cardiology guidelines of the Brazilian Society of Cardiology—2019. Arq Bras Cardiol.

[8] Regitz-Zagrosek V, Roos-Hesselink JW, Bauersachs J (2018). 2018 ESC Guidelines for the management of cardiovascular diseases during pregnancy. Eur Heart J.

[9] Hindricks G, Potpara T, Dagres N, et al. 2020 ESC Guidelines for the diagnosis and management of atrial fibrillation developed in collaboration with the European Association of Cardio-Thoracic Surgery (EACTS). Eur Heart J. 2020;1–125.

[10] Williams B, Mancia G, Spiering W (2018). 2018 ESC/ESH guidelines for the management of arterial hypertension. Eur Heart J.

[11] Brignole M, Auricchio A, Baron-Esquivias G (2013). 2013 ESC Guidelines on cardiac pacing and cardiac resynchronization therapy: the Task Force on cardiac pacing and resynchronization therapy of the European Society of Cardiology (ESC). Developed in collaboration with the European Heart Rhythm Association (EHRA). Eur Heart J.

[12] Mond HG, Proclemer A (2011). The 11th world survey of cardiac pacing and implantable cardioverter-defibrillators: calendar year 2009—a World Society of Arrhythmia’s project. Pacing Clin Electrophysiol.

[13] Knuuti J, Wijns W, Saraste A (2020). 2019 ESC guidelines for the diagnosis and management of chronic coronary syndromes. Eur Heart J.

[14] Collet JP, Thiele H, Barbato E (2020). ESC Guidelines for the management of acute coronary syndromes in patients presenting without persistent ST-segment elevation. Eur Heart J..

[15] Ibanez B, James S, Agewall S (2017). 2017 ESC Guidelines for the management of acute myocardial infarction in patients presenting with ST-segment elevation. Rev Esp Cardiol.

[16] Ponikowski P, Voors AA, Anker SD (2016). 2016 ESC Guidelines for the diagnosis and treatment of acute and chronic heart failure: The Task Force for the diagnosis and treatment of acute and chronic heart failure of the European Society of Cardiology (ESC)Developed with the special contribution of the Heart Failure Association (HFA) of the ESC. Eur Heart J.

[17] Hill L, Prager Geller T, Baruah R (2020). Integration of a palliative approach into heart failure care: a European Society of Cardiology Heart Failure Association position paper. Eur J Heart Fail.

[18] Baumgartner H, Falk V, Bax JJ (2017). 2017 ESC/EACTS Guidelines for the management of valvular heart disease. Eur Heart J.

[19] Cosentino F, Grant PJ, Aboyans V (2020). 2019 ESC Guidelines on diabetes, pre-diabetes, and cardiovascular diseases developed in collaboration with the EASD. Eur Heart J.

[20] Mach F, Baigent C, Catapano AL (2020). 2019 ESC/EAS Guidelines for the management of dyslipidaemias: lipid modification to reduce cardiovascular risk. Eur Heart J.

